# Could the Construct of Modern-Type Depression Predict Internet Gaming Disorder in Italian Video Gamers? A Case–Control Study

**DOI:** 10.3390/brainsci14010048

**Published:** 2024-01-03

**Authors:** Laura Orsolini, Giulio Longo, Silvia Bellagamba, Takahiro A. Kato, Umberto Volpe

**Affiliations:** 1 Unit of Clinical Psychiatry, Department of Clinical Neurosciences/DIMSC, Polytechnic University of Marche, 60126 Ancona, Italy; giulio.longo1996@gmail.com (G.L.); silvibella95@gmail.com (S.B.); u.volpe@staff.univpm.it (U.V.); 2 Department of Neuropsychiatry, Graduate School of Medical Sciences, Kyushu University, Fukuoka 812-8582, Japan; kato.takahiro.015@m.kyushu-u.ac.jp

**Keywords:** Internet Gaming Disorder (IGD), Modern-Type Depression (MTD), post-modern depression, youth mental health, technopathies, video-gamers

## Abstract

A new postmodern depression type, named “Modern-Type Depression” (MTD), is emerging in Western countries. MTD is often underdiagnosed, mainly due to potentially higher comorbidity with technology-based addictions, including Internet Gaming Disorder (IGD). However, the definition of the relationship between MTD and IGD is still controversial, as few data have been published thus far. In particular, there are no data specifically investigating the prevalence of MTD within Italian young subjects with IGD, as well as their mutual association. Hence, within the SWATCH (Social Withdrawal and TeCno-mediated mental Health issues) project, our study aimed to identify the prevalence of MTD in a sample of Italian young adults who play video games by providing a clinical characterization of MTD within a group of IGD individuals (IGD+) versus a group without IGD (IGD−) who play video games. Our cross-sectional case–control study recruited a sample of 543 Italian young video-gaming players (aged 18–35) from the larger SWATCH database, stratified as IGD+ versus IGD−. Subjects were administered the 22-item Tarumi’s Modern-Type Depression Trait Scale (TACS-22), the Motives for Online Gaming Questionnaire (MOGQ), and the Internet Gaming Disorder Scale-Short Form (IGDS9-SF). Around 21.7% of the total sample was represented by MTD individuals, while within the IGD sample, around 34% of subjects had MTD. Within the MTD group, significantly higher scores at IGDS-9SF (*p* < 0.001), MOGQ “Escape from reality” (*p* < 0.001), “Fantasy” (*p* < 0.001), and MOGQ total score (*p* = 0.003) were found compared to MTD−. According to the multivariate regression model, controlled for sex and age, higher scores in the TACS-22 were positively predicted by the total score of IGDS9-SF (*p* = 0.003), the MOGQ “Escape from Reality” subscale (*p* = 0.014), and MOGQ “Fantasy” (*p* = 0.011), and negatively predicted by the MOGQ “Competition” subscale (*p* = 0.035) [F (4538) = 17.265; *p* < 0.001]. Our findings suggested that MTD displays a strong association with IGD. Video-gaming players who do not have IGD appear to be less prone to MTD; this suggests that further studies could be carried out to specifically investigate whether pathological use of video games could also be determined by the presence of MTD.

## 1. Introduction

A new psychopathological dimension named “Modern-Type Depression” (MTD), firstly described as a Japanese/culture-bound condition around the early 2000s [1,2], is rapidly spreading in Western countries [3]. MTD appears to be particularly experienced by the digital natives’ generation, as a new phenotypic manifestation of so-called “*postmodern depression*” [2,3,4]. MTD (translated from the Japanese terms “*Shin-gata*” and “*Gendai-gata utsu-byo*”) is characterized by a depressive status, experienced specifically in stressful work and/or school situations and/or activities, accompanied by a rapid symptomatology improvement or full disappearance when the individual stays retired in home-based settings [2]. MTD differs from other types of depression specifically described in Japan but also in Western countries, including “*taikyaku shinkei-sho*” (i.e., isolation neurosis-type depression), “*tohi-gata utsu-byo*” (i.e., avoidant-type depression), and “*mizyuku-gata utsu-byo*” (i.e., immature-type depression) [1]. Kato et al. [2] more recently proposed a set of MTD diagnostic criteria (Table 1).

However, despite these proposed criteria, most individuals with a typical MTD phenotypic manifestation are more frequently diagnosed by clinicians as having a predominant depressive affective temperament, a dysthymic mood disorder, a personality disorder (i.e., narcissistic, avoidant, schizoid), or as subjects who are not affected by any psychiatric condition (e.g., those without the will to engage in working and/or school activities), rather than being properly identified as MTD subjects [5]. Moreover, as MTD is frequently comorbid with technology-based addictions, including Internet Gaming Disorder (IGD) [6,7,8], it has been assumed to be an underestimated diagnosis, particularly in Western countries, because it is frequently masked by these addictive behaviours [3]. Indeed, one could argue that Internet, social media, and online video gaming could act as means to escape from an unacceptable reality by facilitating the individual’s isolation from real life, allowing them to live in a more satisfactory digital world, which could indeed predispose them to the onset of MTD [8]. Contrarily, digital tools and Internet-based activities may potentially represent a sort of coping strategy for MTD individuals, acting as the only residual elements of any (lost in-person) social contact [3,8]. However, the type of relationship between MTD and techno-addictions, including IGD, is still controversial, as few data have been published thus far. In particular, no data specifically investigating the prevalence of MTD within Italian young subjects with IGD and their reciprocal association have been published thus far. Therefore, within the SWATCH (Social Withdrawal and TeCno-mediated mental Health issues) project, a cross-sectional case–control study was conducted here to specifically investigate the presence of MTD within a sample of Italian video gamers with and without a confirmed diagnosis of IGD, in order to evaluate the role of MTD in the onset and/or maintenance of IGD. More specifically, the main objectives of our study were (a) identifying the prevalence of MTD in a sample of Italian young adults who play video games, and, (b) providing a clinical characterization of MTD within a group of IGD individuals (IGD+) versus the group who play video games without IGD (IGD−), in order to better understand how MTD may play a role in the onset of IGD. Exploratory outcomes include exploring which subcategories of video gaming players (in terms of motivations in video gaming) are more represented within MTD subjects.

## 2. Materials and Methods

### 2.1. Study Design and Recruitment Strategies

The study was carried out by recruiting a sub-sample of Italian young people (aged 18–35), coming from the larger database of the SWATCH study, during the timeframe of January–February 2022. Only subjects who declared that they play video games (both online and/or offline) were included in the study. No selection of specific video games to be played have been performed in order to consider the participants eligible inclusion in the study. All subjects were also screened for other previous and/or current concomitant psychiatric disorders and properly excluded, in order to obtain a sample without comorbid psychiatric disorders. All subjects who complained of psychological (not psychiatric) distress were indeed included in the study. Then, the sample was split in two groups: subjects with IGD (IGD+) and subjects without IGD (IGD−), by using the Internet Gaming Disorder Scale-Short Form (IGDS9-SF) [9]. The WHO sample size calculator [10] was used to establish the minimum required sample size by setting the confidence level as 99%, the anticipated population proportion at 0.5, an α error of 0.05, a power of 80%, and considering all variables to be entered in the multivariable analysis. Hence, a minimum sample size of 400 was established a priori, in order to obtain an effect size of at least >0.6. All participants gave informed consent to take part in the study. The study was conducted in accordance with the ethical principles outlined in the Declaration of Helsinki and according to the guidelines for Good Clinical Practice (GCP), following the approval by the local Institutional Review Board. The CHERRIES checklist was followed in reporting the findings [11].

### 2.2. Measurements

An *ad hoc* case report form (CRF) was developed to collect respondents’ socio-demographic and clinical characteristics, including participants’ age, sex, occupational and marital status, average daily number of gaming hours, and type of video gaming activity.

The IGDS9-SF [9] consists of 9 items, corresponding to the 9 diagnostic criteria of the DSM-5 [12] for Internet Gaming Disorder. Each item is rated from 1 (Never) to 5 (Very often). The scale defines the severity and consequences of IGD, assessing the patient’s online and offline gaming activity in the past 12 months. The scale investigates the following domains: worry, tolerance, withdrawal, reduction/interruption, loss of interest, continued use, deception, escape, and conflict. A cut off ≥ 21 was established to distinguish IGD players from non-IGD players. In the validation study conducted by Monacis et al. [9], a Cronbach’s α index of 0.96 with excellent internal consistency was reported. In our study, the Cronbach’s α of the IGDS9-SF similarly showed good internal reliability (α = 0.838). 

The Motives for Online Gaming Questionnaire (MOGQ) [13] consists of 27 items, each of them scoring from 1 (Never) to 5 (Almost always/always), and assesses the main determinants/motivations underpinning online gaming. The scale has the following 7 subscales: socialization, escape from reality, competition, coping, skill development, fantasy and recreation. The MOGQ displays good psychometric properties (χ^2^ = 1909.0, df = 299, *p* = 0.107; CFI = 0.939; TLI = 0.928; RMSEA = 0.052 [0.049–0.054]; Cfit = 0.0107; SRMR = 0.046). In our study, the MOGQ displayed good internal reliability (Cronbach’s α = 0.934). 

The 22-item Tarumi’s Modern-Type Depression Trait Scale (TACS-22) [2,14] is a questionnaire designed to identify MTD. The scale consists of 22 items and three subscales (social role avoidance, low self esteem, and malaise), and each is given a score from 0 (Disagree) to 4 (Agree). The cutoff of psychopathological interest has been established to be ≥54 [2,14]. The Cronbach’s α of the TACS-22 in our study showed good internal reliability (α = 0.829).

### 2.3. Statistical Analysis

Descriptive statistics were used in order to describe the socio-demographic and clinical characteristics of the sample. After verifying the normality of the distribution of the continuous variables using skewness, kurtosis and the Kolmogorov–Smirnov test, the Levene test, parametric (independent samples Student’s *t*-test and two-way tailored analysis of variance [ANOVA]) and nonparametric statistical tests (U-Mann–Whitney and Kruskal–Wallis tests) were used, when appropriate, to establish the equality of variances. Normally distributed continuous variables were represented using the average mean and standard deviation (SD), when normally distributed, or the median and 95% Confidence interval (95% CI) when not normally distributed. Categorical variables are summarized as frequency (*n*) and percentage (%), whilst continuous variables are means (plus standard deviation (SD)). Categorical variables were compared using the χ^2^ test, as well as all socio-demographic variables between the following paired groups: IGD versus non-IGD, and MTD versus non-MTD. Bivariate Pearson’s correlations have been used to investigate potential relationships between the IGDS9-SF total score and TACS-22 total scores and subscales, as well as between the TACS-22 total score and subscales and MOGQ and its subscales. Linear regression analysis was performed by firstly considering IGD (as measured by the IGDS9-SF) as a primary outcome in order to evaluate whether premorbid MTD personality traits and their subscales (as measured by TACS-22) could be a predictor of a higher risk of developing IGD in a young sample of video game players. Similarly, a stepwise multivariate linear regression model was used to investigate video gaming-related predictors of MTD (as measured by TACS-22), including the following independent variables: MOGQ and its subscales, IGDS9-SF, and all collected socio-demographic features of the sample. For all analyses, the level of statistical significance was set at *p* < 0.05 (two-tailed). All statistical analyses were performed using the software Statistical Package for Social Science (SPSS) version 27.0 for MacOS (IBM SPSS Statistics, Chicago, IL, USA).

## 3. Results

### 3.1. Socio-Demographic Characteristics of the Sample

A sample of 543 participants was included for the study, among those who declared that they play video games (Table 2).

Around 78% of the sample (*n* = 424) declared that they play video games both online and offline. Most participants declared that they play video games both during the week and during the weekend (*n* = 445; 42.7%). A third of the sample declared usually playing video games for more than 7 h a week. The participants’ mean age was 21.7 (SD = 3.4), without sex-based differences (*p* = 0.627). Within the sub-sample of video gamers who declared a unique preferred video game (*n* = 424), around 40.1% of the sample (*n* = 218) declared that they preferentially played shooting video games, followed by 35.9% (*n* = 195) who preferred strategy-based video games, 27.4% (*n* = 149) who preferred adventure-type video games, and 25.8% who preferred action-type video games (*n* = 140). Around two-thirds of the sample is represented by males (*n* = 328; 60.5%), while most participants were university students (*n* = 427; 77.9%) (Table 2). 

### 3.2. Psychopathological Characteristics of the Sample

The mean score at IGDS9-SF was 15.3 (SD = 5.7), without age-based differences (*p* = 0.504), with subjects with a confirmed diagnosis of IGD making up around 17.3% of the total sample (IGD+). Female sex was a protective factor for the onset of IGDS9-SF in our sample (OR = 1.7) (Table 2). Most IGD+ individuals declared that they play video games both online and offline (79.8%), and more significantly, that they play both during the week and the weekend (*p* = 0.040). There were no differences in the type of video game played between subjects with IGD and subjects without IGD. The mean total score in MOGQ was 64.0 (SD = 20.9), with significant sex-based differences for each MOGQ subscale except for Escape from Reality in MOGQ (*p* = 0.201) (Table 3).

For all other subscales, males displayed significantly higher scores (with *p* < 0.001 for “Competition”, “Management Skills”, “Ability Development” and “Recreation”, and *p* = 0.017 for “Socialization” and *p* = 0.021 for “Fantasy”). Among those players who claimed to prefer online video gaming, significantly higher scores in MOGQ “Socialization” were found compared to those with an offline preference (*p* = 0.002). Among those players who claimed to prefer offline video gaming, we found significantly lower scores in MOGQ “Escape from Reality”, “Competition”, “Management Skills”, “Ability Development”, and “Recreation” compared to those who declared a preference for online gaming (all with *p* < 0.001). 

### 3.3. Modern-Type Depression Characterization in Our Sample in Relation to Video Gaming and Internet Gaming Disorder

The mean score at the TACS-22 was 43.4 (SD = 14.2), without any age-based differences (*p* = 0.955), and with around 21.7% of the total sample represented by MTD individuals (Table 4). Meanwhile, the rate of MTD subjects was found to be significantly higher within the IGD+ group, compared to IGD− subjects (34% and 19.2%, respectively) (χ^2^ = 10.130; *p* = 0.001) (OR = 2.2). When analyzing the factor of video gaming, significantly higher scores in the IGDS-9SF (*p* < 0.001), and in MOGQ “Escape from reality” (*p* < 0.001), “Fantasy” (*p* < 0.001), and MOGQ total score (*p* = 0.003) were found within the MTD+ group, compared to the MTD−group (Table 5). According to the type of preferred video game, significantly lower TACS-22 scores were found among those individuals who preferentially played shooting video games (*p* = 0.010). Meanwhile, significantly higher scores in TACS-22 were found among those video gamers who preferred to play arcade video games (*p* = 0.009), fighting games (*p* = 0.029), or role-playing games (*p* = 0.034).

Significantly higher scores in MOGQ “Management Skills” (*p* = 0.001) were observed among MTD− compared to MTD+ subjects. Within the MTD group, no significant differences were observed regarding preference for online and/or offline video gaming (*p* = 0.083). The absence of an IGD represents a protective factor for the presence of MTD (OR = 0.563; 95%CI = 0.401–0.790). IGD subjects display significantly higher scores in TACS-22 (*p* < 0.001), with higher IGDS9-SF scores being significantly predicted by higher TACS-22 scores [B = 0.106 (95%CI = 0.073–0.139); *p* < 0.001]. Finally, according to the multivariate regression model, controlled for sex and age, higher scores at TACS-22 were positively predicted by the total score of IGDS9-SF (B = 0.369, 95%CI = (0.123–0.615), *p* = 0.003), the MOGQ “Escape from Reality” subscale (B = 0.450, 95%CI = (0.091–0.810), *p* = 0.014), and MOGQ “Fantasy” (B = 0.493, 95%CI = (0.113–0.873), *p* = 0.011), and were negatively predicted by the MOGQ “Competition” subscale (B = −0.299, 95%CI = ((−0.576)–(−0.022), *p* = 0.035) [F (4538) = 17.265; *p* < 0.001] (Table 6) (Supplementary Table S1).

## 4. Discussion

### 4.1. Summary of the Main Findings of the Study

To the best of our knowledge, this is the first study investigating MTD in an Italian sample recruited within a cohort of young adults who currently play video games, by comparing those who display an IGD and those who play video games in a non-pathological way. Only one Japanese study carried out by Tateno and Kato has previously investigated MTD in the context of technology-based addictions, even though the authors explored the association between MTD and smartphone addiction [15]. Their findings clearly demonstrated that a higher severity of smartphone addiction was significantly associated with higher scores in TACS-22, which was used to evaluate the premorbid personality traits of MTD [15]. Our findings found an overall prevalence rate of 21.7% of MTD in our sample of video game players. When analyzing the MTD rates by splitting the total sample into IGD+ subjects versus IGD− subjects, a significant higher percentage of MTD subjects were found within the IGD+ group, compared to IGD− subjects (OR = 2.2). Indeed, one could preliminarily argue that IGD subjects could be more significantly represented by MTD, although IGD subjects compared to non pathological video gamers could significantly differ in many psychological features that could be directly or indirectly implicated in the aetiopathogenesis of MTD. Therefore, further studies should adequately assess and investigate which other potential psychological variables could potentially influence (or not) the relationship between MTD and IGD, before drawing up definitive conclusions.

In our study, we found a percentage of 17.3% of IGD subjects, which is higher compared to previous published Italian population-based studies [16,17,18,19]. However, one could argue that the highest percentage in our study could be explained by the high selectivity of our sample, only represented by video gaming players. Moreover, our sample is mainly represented by males, in line with previously published studies [16,17,20]. In our study, significantly higher IGDS9-SF scores have been found among MTD individuals, suggesting a possible association between IGD and MTD. In particular, those video gaming players who do not have IGD appear to be less prone to displaying MTD, which could suggest that pathological video game use could potentially be a risk factor for MTD. Indeed, in our study, MTD individuals did not appear to have a preference for online versus offline video gaming modalities. However, our findings clearly found that higher TACS-22 scores are significantly predicted by higher IGDS9-SF. Moreover, our multivariate regression analysis clearly reported that video game players who declared the need to ‘escape from reality’ (as measured by the MOGQ subscale “Escape from reality”) or the need to activate their ‘fantasy’ because of their feelings of boredom (as measured by the MOGQ subscale “Fantasy”) as their main motivations for online video gaming are those who are more likely to display MTD. Meanwhile, those video game players who declared the need to experience ‘competition’ (as measured by the MOGQ subscale ‘competition’) as their main motivation for playing online video games are those less likely display MTD. Furthermore, when we analyze the preferred type of video game, we found significant higher TACS-22 scores among those subjects who predominantly play to role-play, arcade, or fighting video games, and significantly lower scores in TACS-22 among those who claimed to preferentially play shooting video games. Indeed, these findings are also in line with findings regarding the abovementioned main motivators of online video gaming. Demetrovics et al. [13] found video gamers had a motivation to escape in order to avoid real life problems and difficulties, and that fantasy games involve trying new sensations, and seeking activities/identities in virtual game worlds that are not possible in everyday life (such as virtual avatars). Exploring the motivations associated with IGD allows clinicians to better characterize and treat the psychopathological dimensions underpinning pathological online video gaming [13,21]. For instance, escape from reality has been strongly associated with IGD due to efforts to avoid real-life difficulties, and fantasy has been associated with feelings of boredom [22,23,24,25,26].

Moreover, one could argue that whereas video gaming players may manifest more narcissistic personality traits, the spirit of competition, and the need to demonstrate their value and competencies, MTD does not seem to be significantly represented. That said, in those individuals in which there is the need to escape from an unacceptable and non-incentivizing reality, such as that described by MTD individuals, using video games (until in a pathological manner) and preferring a particular type of video game (such as role-play and arcade games, which could facilitate an ‘escape from an unacceptable reality and the build of a new more acceptable and winning virtual identity’) could be predicted by premorbid MTD traits. Moreover, one could argue that the element of boredom could also represent a mediator in the relationship between MTD and the emergence of IGD, particularly in those individuals in which the main motivator for online video gaming is the need to activate their fantasy and experience new sensations/novel activities. Indeed, previous studies have clearly investigated the role of boredom in mediating the association between depressive symptomatology and the onset of technology-based addictions [27,28,29].

### 4.2. Limitations and Strengths of the Study

Despite these promising findings, our study indeed presents several limitations. Firstly, the sample is highly selective, being represented by only young adults (aged 18–35) who play video games, mainly males, university students, and unemployed individuals. Moreover, a percentage of video gamers in our sample did not clearly declare an unique preference regarding type of video game, therefore limiting the interpretation of our findings about the role of MTD in preference for a specific type of video game within the sample of IGD+ versus IGD− individuals. Indeed, the sub-analysis within only the sample of video gamers who clearly declared a unique preference for one type of video game allowed us to find a clear trend in the type of video games preferred by subjects who displayed higher scores in TACS-22, thereby suggesting a possible trend in the choice of the type of video game in this population, which should be confirmed with further larger longitudinal studies. Hence, our findings could be extremely useful in finding preventive strategies for non-pathological video gaming players and for personalizing the treatment of IGD individuals. Another important limitation is represented by the fact that our sample is not fully representative of the general population, being only represented by young Italian video gamers. Moreover, our sample is not representative of a sample of subjects with other comorbid psychiatric disorders, as we excluded a priori those subjects with a previous and/or a current psychiatric disorder. However, our sample is indeed represented by young subjects with psychological (not psychiatric) distress; this could represent a strength as it could potentially include and reflect the current panorama of adolescent mental health within generation Z, thus potentially helping us to identify at-risk youths early. Further studies should also investigate MTD within the general population by also stratifying the sample according to the age and sex, and not only selecting video gamers. Moreover, our study did not investigate MTD dimensions related to other techno-based addictions (e.g., smartphone addiction, Internet addiction, and so forth). Hence, it was not possible to compare our findings with the only study currently published [15]. Furthermore, our study screened individuals by using a self-report scale for MTD (i.e., TACS-22), which has been developed by Kato et al. [2] as a tool to evaluate the premorbid personality of Tarumi’s MTD. Although TACS-22 has been validated in the Italian population [14], it was specifically developed for the Japanese population, hence we should also consider the need to cross-culturally adapt the scale and evaluate whether Japanese MTD is similar to Italian MTD [3]. Contrarily, with our sample being highly selective for video gamers, we had the opportunity to compare MTD among pathological versus non-pathological online video gamers. Finally, the cross-sectional nature of our study does not allow us to draw definitive conclusions regarding the causal relationship between MTD and IGD (i.e., establishing whether an individual with a premorbid MTD will more likely develop IGD among video gamers, or whether having a IGD may facilitate the emergence of a MTD status). Therefore, further longitudinal studies should be conducted to better characterize this new psychopathological dimension in the Italian population, as well as its role in the onset and/or maintenance of IGD and other techno-mediated psychopathologies/addictions.

### 4.3. Future Research Directions

Overall, given the potential clinical implications of exploring the new theoretical construct of MTD within technology-based addictions, in terms of preventive strategies and new therapeutic approaches, useful future research directions should be explored in order to clinically characterize this emerging psychopathological dimension across Western countries by clearly identifying if any differences exist across different cultures in the clinical manifestation as well as in the pathogenesis of MTD, considering the variable presence/absence of a technology addiction as a potential risk factor or rather a consequence of a premorbid MTD condition. MTD should be carefully investigated regarding its potential role in the onset and/or maintenance of technology-based addiction, including in facilitating the shift from non-pathological video gaming to a pathological habit (namely, IGD). Another issue to be carefully considered is the role that MTD could play specifically within this sample of Italian young people, and to evaluate whether problematic use of technology may act as an accelerator or a coping strategy for these individuals as they try to manage/cope with boredom and/or affective/emotional emptiness, which characterize current postmodern (aka ‘Modern-Type’) depression [29,30].

## 5. Conclusions

Overall, the new construct of the MTD, as conceptualized by Kato et al. [1,2,5], should more likely be interpreted considering the new form of adjustment-like reactions of youngsters to current postmodern/liquid society, which is more likely to contain an individualistic performance-based system than collectivistic and socially mutual society and community. Within this context, MTD and its possible relationship with current techno-based addictions could be better explained and understood if we consider the multifaceted opportunities offered by the web, the Internet, and the world of video gaming in terms of alternative virtual realities/worlds in which anyone can potentially become more successful, and wear a more successful and ‘socially acceptable’ mask. The chance to escape from real life and to build alternative identities able to cope with and overcome social pressure and move towards competence and performance could offer an attractive and engaging platform to many youngsters. Therefore, there is a need to culturally understand and transfer preliminary findings coming from Japanese culture to our Italian context in order to identify and investigate this new emerging and dramatic psychopathological dimension; in doing so, we may implement more tailored preventive and innovative treatment strategies, and accurately identify at-risk young people.

## Figures and Tables

**Table 1:** Diagnostic criteria for Modern-Type Depression (from Kato et al., 2017 [5]).

An overt appearance of depressive mood, which is based on a belief that one is clinically depressed. One (or more) of the following symptoms must be present, and must be confirmed by informants (e.g., family members, colleagues): The individual has had or expressed a desire to be excused or spared from duties or responsibilities (e.g., school, work) because of depression; The individual’s overall functioning worsens during work or school, whereas it is relatively maintained at other times. Three (or more) of the following five characteristics (i.e., personality trait, behavioral pattern, interpersonal pattern) must be present, and must be confirmed by informants (e.g., family members, colleagues): Has never been diligent; An avoidance/hatred of hierarchies and ranks in society; A preference to exist without social roles; An extrapunitive type; A vague sense of omnipotence. The symptoms cause clinically significant distress or impairment in social, occupational, or other important areas of functioning. The symptoms are not attributable to the physiological effects of a substance or another medical condition.

Note: The presence of three or more of the characteristics in (3) is thought to indicate the temperament of MTD. However, having this temperament is not equated with a clinical case of MTD.

**Table 2:** Socio-demographic characteristics of the sample based on IGD.

	Total Sample	Not-IGD	IGD	*p*-Value
**Age**				
Males	21.8 ± 3.6	21.7 ± 3.5	21.8 ± 3.6	*p* = 0.798
Females	21.6 ± 3.3	21.6 ± 3.3	21.8 ± 2.9	*p* = 0.393
**Sex**				
Males	328 (60.4%)	257 (57.2%)	71 (75.5%)	χ^2^ = 10.876 *p* * < 0.001
Females	215 (39.6%)	192 (42.8%)	23 (24.5%)	
**Education**				
Middle school certificate	16 (3.0%)	12 (2.7%)	4 (4.3%)	χ^2^ = 1.802 *p* = 0.614
High school diploma	378 (69.7%)	312 (69.6%)	66 (70.2%)	
Degree	137 (25.2%)	116 (25.9%)	21 (22.3%)	
Post-graduate diploma	11 (2.0%)	8 (1.8%)	3 (3.2%)	
**Working status**				
High school student	11 (2.0%)	9 (2.0%)	2 (2.1%)	χ^2^ = 1.672 *p* = 0.892
University student	423 (77.9%)	351 (78.3%)	71 (75.5%)	
Working student	38 (7.0%)	32 (7.1%)	6 (6.5%)	
Full time worker	50 (9.2%)	40 (8.9%)	10 (10.6%)	
Part time worker	7 (1.3%)	6 (1.3%)	1 (1.1%)	
Unemployed	14 (2.6%)	10 (2.2%)	4 (4.3%)	
**Type of video playing**				
Only online	46 (8.5%)	37 (8.2%)	9 (9.6%)	χ^2^ = 0.871 *p* * = 0.651
Only offline	73 (13.4%)	63 (14.0%)	10 (10.6%)	
Online and offline	424 (78.1%)	349 (77.7%)	75 (79.8%)	
**Video game playing activity**				
During the week	47 (8.7%)	42 (9.5%)	5 (5.3%)	χ^2^ = 6.408 ***p* = 0.041**
During the weekend	46 (8.6%)	43 (9.7%)	3 (3.2%)	
Both	445 (82.7%)	359 (80.9%)	86 (91.5%)	
**Time spent playing weekly**				
Less than 1 h	81 (15.0%)	76 (17%)	5 (5.3%)	χ^2^ = 28.040 ***p* < 0.001**
1–3 h	142 (26.2%)	126 (28.2%)	16 (17.0%)	
4–6 h	120 (22.2%)	103 (23.0%)	17 (18.1%)	
More than 6 h	198 (36.6%)	142 (31.8%)	56 (59.6%)	

Significant *p*-values are in bold. IGD: Internet Gaming Disorder. * Fisher’s exact test.

**Table 3:** Psychopathological features of the sample based on Internet Gaming Disorder.

	Total Sample	Not-IGD	IGD	*p*-Value
TACS-22 Total score	43.3 ± 14.0	42.3 ± 14.2	48.4 ± 11.6	***p* < 0.001**
MOGQ Socialization	8.4 ± 3.7	8.0 ± 3.6	10.2 ± 3.7	***p* < 0.001**
MOGQ Escape	8.2 ± 4.5	7.3 ± 3.9	12.6 ± 4.7	***p* < 0.001**
MOGQ Competition	9.7 ± 4.3	9.3 ± 4.2	12.0 ± 4.2	***p* < 0.001**
MOGQ Coping	9.5 ± 3.7	9.0 ± 3.5	11.8 ± 3.7	***p* < 0.001**
MOGQ Skill development	9.1 ± 4.8	8.8 ± 4.7	11.0 ± 5.0	***p* < 0.001**
MOGQ Fantasy	7.0 ± 3.9	6.5 ± 3.6	9.5 ± 4.5	***p* < 0.001**
MOGQ Recreation	12.0 ± 3.5	12.0 ± 3.5	12.4 ± 3.2	*p* = 0.526

MOGQ: Motives for Online Gaming Questionnaire; TACS-22: 22-item Tarumi’s Modern-Type Depression Trait Scale. Significant *p*-values are in bold.

**Table 4:** Socio-demographic characteristics of the sample based on Modern-Type Depression.

	Total Sample	Not-MTD	MTD	*p*-Value
**Age**				
Males	21.8 ± 3.6	21.7 ± 3.5	21.8 ± 3.7	*p* = 0.547
Females	21.6 ± 3.3	21.6 ± 3.3	21.6 ± 3.1	*p* = 0.901
**Sex**				
Males	328 (60.4%)	272 (64.2%)	55 (47.4%)	χ^2^ = 10.682 ***p* = 0.001**
Females	214 (39.6%)	152 (35.8%)	61 (52.6%)	
**Education**				
Middle school certificate	16 (3.0%)	11 (2.6%)	5 (4.2%)	χ^2^ = 3.055 *p* = 0.383
High school diploma	378 (69.7%)	291 (68.6%)	87 (73.7%)	
Degree	137 (25.2%)	113 (26.7%)	24 (20.3%)	
Post-graduate diploma	11 (2.0%)	9 (2.1%)	2 (1.7%)	
**Working status**				
High school student	11 (2.0%)	9 (2.1%)	2 (1.7%)	χ^2^ = 4.950 *p* = 0.422
University student	423 (77.9%)	326 (76.9%)	96 (81.4%)	
Working student	38 (7.0%)	31 (7.3%)	7 (5.9%)	
Full time worker	50 (9.2%)	43 (10.1%)	7 (5.9%)	
Part time worker	7 (1.3%)	4 (0.9%)	3 (2.5%)	
Unemployed	14 (2.6%)	11 (2.6%)	3 (2.5%)	
**Type of video game playing**				
Only online	46 (8.5%)	38 (9.0%)	8 (6.8%)	χ^2^ = 2.920 *p* = 0.232
Only offline	73 (13.4%)	52 (12.3%)	21 (17.8%)	
Online and offline	424 (78.1%)	334 (78.8%)	89 (75.4%)	
**Video game playing activity**				
During the week	47 (8.8%)	37 (8.8%)	10 (8.6%)	χ^2^ = 0.228 *p* = 0.892
During the weekend	46 (8.4%)	34 (8.1%)	11 (9.5%)	
Both	445 (82.8%)	349 (83.1%)	95 (81.9%)	
**Time spent playing weekly**				
Less than 1 h	81 (15.0%)	70 (16.5%)	11 (9.5%)	χ^2^ = 4.386 *p* = 0.223
1–3 h	142 (26.2%)	107 (25.3%)	35 (30.2%)	
4–6 h	120 (22.2%)	91 (21.5%)	29 (25.0%)	
More than 6 h	198 (36.6%)	155 (36.6%)	41 (35.3%)	

Significant *p*-values are in bold. MTD: Modern-Type Depression.

**Table 5:** Psychopathological features of the sample based on the presence/absence of the premorbid Modern-Type Depression personality trait.

	Total Sample	Not-MTD	MTD	*p*-Value
IGDS9SF Total score	15.3	14.7 ± 5.0	17.5 ± 7.3	***p* < 0.001**
MOGQ Socialization	8.4 ± 3.7	8.3 ± 3.8	8.7 ± 3.6	*p* = 0.365
MOGQ Escape from reality	8.2 ± 4.5	7.6 ± 4.0	10.4 ± 5.4	***p* < 0.001**
MOGQ Competition	9.7 ± 4.3	9.7 ± 4.3	9.9 ± 4.6	*p* = 0.605
MOGQ Coping	9.5 ± 3.7	9.2 ± 3.5	10.6 ± 4.2	***p* < 0.001**
MOGQ Skill development	9.1 ± 4.8	9.1 ± 4.6	9.4 ± 5.3	*p* = 0.479
MOGQ Fantasy	7.0 ± 3.9	6.5 ± 3.4	8.9 ± 4.9	***p* < 0.001**
MOGQ Recreation	12.0 ± 3.5	12.1 ± 3.5	11.8 ± 3.6	*p* = 0.328
MOGQ Total score	64.0 ± 20.9	62.4 ± 19.7	69.7 ± 23.8	***p* < 0.001**

MOGQ: Motives for Online Gaming Questionnaire; TACS-22: Tarumi’s Modern-Type Depression Trait Scale. Significant *p*-values are in bold.

**Table 6:** Multiple linear regression with TACSS-22 total score (as a dependent variable).

	B	SE	β	t	*p*-Value	95%IC Lower Limit	95%IC Upper Limit	Tolerance	VIF
(*constant*)	33.475	1.862		17.979	<0.001	29.817	37.132		
**MOGQ “Escape from reality” subscale**	0.450	0.183	0.144	2.459	**0.014**	0.091	0.810	0.481	2.080
**MOGQ “Fantasy” subscale**	0.493	0.194	0.137	2.547	**0.011**	0.113	0.873	0.568	1.762
**MOGQ “Competition” subscale**	−0.299	0.141	−0.093	−2.120	**0.035**	−0.576	−0.022	0.863	1.159
**IGDS9-SF total score**	0.369	0.125	0.149	2.942	**0.003**	0.123	0.615	0.643	1.555

SE: Standard Error; TACS-22: Tarumi’s Modern-Type Depression Trait Scale; MOGQ: Motives for Online Gaming Questionnaire; IGDS9-SF: Internet Gaming Disorder Scale 9-Short Form. Significant *p*-values are in bold.

## Data Availability

The data presented in this study are available on request from the corresponding author. The data are not publicly available due to ethical restrictions.

## Supplementary Materials

The following supporting information can be downloaded at: https://www.mdpi.com/article/10.3390/brainsci14010048/s1, Table S1: Supplementary File to Table 6. Multiple Linear Regression with TACSS-22 total score (as dependent variable)—other not significant variables excluded by the model.
