# Supplementary material for: Could the Construct of Modern-Type Depression Predict Internet Gaming Disorder in Italian Video Gamers? A Case–Control Study
Source: Brain Sci. 2024 Jan 3;14(1):48. doi: 10.3390/brainsci14010048 (PMC10813306; doi:10.3390/brainsci14010048)
Supplement: Supplementary file 1 [file brainsci-14-00048-s001.zip › brainsci-2798056-supplementary.pdf]

**Supplementary File to Table 6.** Multiple Linear Regression with TACSS-22 total score (as dependent variable) - other not significant variables excluded by the model.

| | β | t | p-value | Tolerance | VIF |
|---|---|---|---|---|---|
| **MOGQ "Socialization" subscale** | -0.043 | -0.893 | 0.372 | 0.706 | 1.417 |
| **MOGQ "Coping" subscale** | 0.043 | 0.711 | 0.477 | 0.457 | 2.186 |
| **MOGQ "Ability Development" subscale** | -0.044 | -0.902 | 0.367 | -0.679 | 1.472 |
| **MOGQ "Recreation" subscale** | 0.045 | 0.965 | 0.335 | 0.747 | 1.339 |
| **MOGQ total scale** | -0.018 | -0.185 | 0.853 | 0.181 | 5.518 |

TACS-22: Tarumi's Modern-Type Depression Trait Scale; MOGQ: Motives for Online Gaming Questionnaire.
